# DNA-Based Sensor for Real-Time Measurement of the Enzymatic Activity of Human Topoisomerase I

**DOI:** 10.3390/s130404017

**Published:** 2013-03-25

**Authors:** Lærke Bay Marcussen, Morten Leth Jepsen, Emil Laust Kristoffersen, Oskar Franch, Joanna Proszek, Yi-Ping Ho, Magnus Stougaard, Birgitta Ruth Knudsen

**Affiliations:** 1 Department of Molecular Biology and Genetics, Aarhus University, Aarhus C 8000, Denmark; E-Mails: laerke_bay@hotmail.com (L.B.M.); mortenlethjepsen@gmail.com (M.L.J.); emillk@mb.au.dk (E.L.K.); oskar.franch@post.au.dk (O.F.); 2 Department of Pathology, Aarhus University Hospital, Aarhus C 8000, Denmark; E-Mail: joanna.proszek@gmail.com; 3 Interdisciplinary Nanoscience Center (iNANO), Aarhus University, Aarhus C 8000, Denmark; E-Mail: megan.ypho@inano.au.dk

**Keywords:** real-time activity measurement, DNA sensor, fluorophore-quencher pair, topoisomerase I, cancer treatment, camptothecin

## Abstract

Sensors capable of quantitative real-time measurements may present the easiest and most accurate way to study enzyme activities. Here we present a novel DNA-based sensor for specific and quantitative real-time measurement of the enzymatic activity of the essential human enzyme, topoisomerase I. The basic design of the sensor relies on two DNA strands that hybridize to form a hairpin structure with a fluorophore-quencher pair. The quencher moiety is released from the sensor upon reaction with human topoisomerase I thus enabling real-time optical measurement of enzymatic activity. The sensor is specific for topoisomerase I even in raw cell extracts and presents a simple mean of following enzyme kinetics using standard laboratory equipment such as a qPCR machine or fluorimeter. Human topoisomerase I is a well-known target for the clinically used anti-cancer drugs of the camptothecin family. The cytotoxic effect of camptothecins correlates directly with the intracellular topoisomerase I activity. We therefore envision that the presented sensor may find use for the prediction of cellular drug response. Moreover, inhibition of topoisomerase I by camptothecin is readily detectable using the presented DNA sensor, suggesting a potential application of the sensor for first line screening for potential topoisomerase I targeting anti-cancer drugs.

## 1. Introduction

During recent years vast numbers of DNA-based sensors for optical measurement of enzymatic activities or protein binding, often in real-time, have been presented. These include measurements of helicase-, endonuclease- or repair activities as well as protein-DNA interactions and were achieved by ensemble or single-molecule fluorescence resonance energy transfer (FRET) between two fluorophores or by various fluorophore-quenching strategies [1–7]. Optical sensor systems allow investigation of enzymatic steps otherwise difficult to address using conventional methods as exemplified by the measurement of unpairing of viral DNA ends by retroviral integrases [2] or gate-DNA bending by human topoisomerase IIα [3]. Furthermore, sensors have been designed to allow easy real-time measurement of enzyme activity useful for prognostic, diagnostic or drug testing purposes [4,7].

In the present study we have focused on the development of a DNA-based sensor allowing optical and real-time measurement of the cleavage-religation activity of human topoisomerase I (hTopI). This nuclear enzyme plays an essential function during DNA metabolic processes such as transcription, replication and recombination by regulating the topology of genomic DNA [8,9]. This is accomplished via a catalytic cycle that involves the following reaction steps: (i) non-covalent DNA binding; (ii) cleavage of one strand in the DNA helix leading to the formation of a covalent 3′-phosphotyrosyl cleavage intermediate; (iii) strand rotation during which supercoils are removed by rotation of the cleaved 5′-OH DNA end around the uncut DNA strand; (iv) religation of the generated DNA nick; and (v) enzyme release.

Besides its biological importance, hTopI has also gained considerable clinical interest being the sole cellular target of drugs from the camptothecin (CPT) family [10], which are currently used in systemic treatment of colon, ovarian- and small-cell lung cancers [10–12]. At the molecular level CPTs act by selectively inhibiting the religation step of hTopI catalysis by specifically binding the protein-DNA cleavage intermediate in a well-defined manner that highly depends on the presence of DNA [13–15]. This leads to the accumulation of covalent cleavage intermediates in the genomic DNA, which by collision with DNA tracking processes such as the replication machinery causes DNA fragmentation and ultimately cell death [10]. Consequently, the cytotoxic effect of CPTs correlates directly with the intra-cellular activity level of hTopI and depends on active replication [10,16,17]. This explains the anti-cancer effect of the drugs, since most cancer cells are characterized both by an increased hTopI activity and increased replication rate relative to healthy cells. Another factor determining the effectiveness of cancer treatment with CPTs may be the susceptibility of hTopI towards the drugs. Consistently, point-mutations in the gene expressing TopI that affect drug interaction to the cleavage complexes are well known causes of cellular resistance towards CPTs [10,18,19].

Real-time measurement of the strand rotation step of hTopI catalysis has been achieved previously by the clever manipulation of single DNA molecules using magnetic tweezers [20,21]. However, to our knowledge a real-time sensor for measuring the DNA cleavage-religation steps of the catalysis, which presents the clinically most relevant activities of hTopI (CPT shifts the cleavage-religation equilibrium), has not been reported previously. In the current manuscript we demonstrate optical real-time measurement of these activities of hTopI using a two oligonucleotide-composed hairpin shaped DNA sensor with a quencher-fluorophore pair. Upon reaction with hTopI the quencher and fluorophore becomes separated allowing detection of the light emitted from the fluorophore. This sensor design enabled quantitative measurement of hTopI DNA cleavage-ligation activity using a qPCR machine, which is standard in most research- and many clinical laboratories. Moreover, the sensor was demonstrated to be specific towards hTopI activity even when present in crude biological samples and was suitable for measuring the inhibition of hTopI by CPTs. Hence, in longer terms the presented sensor may provide a relatively fast and easy mean for predicting the drug response of individual patients as well as it may prove valuable for fast high-throughput drug screening programs.

## 2. Experimental Section

### 2.1. Yeast Strains and Construction of hTopI Expression Plasmids

The yeast *Saccharomyces cerevisiae* top1-null strain RS190 was a kind gift from R. Sternglanz (State University of New York, Stony Brook, NY, USA). Plasmid pHT143, for expression of recombinant full-length hTopI, was described previously [22].

### 2.2. Expression and Purification of hTopI and Preparation of Cell Extracts

The plasmid pHT143 was transformed into the *S. cerevisiae* strain RS190. The cells were grown, and hTopI expression was induced as described by Björnsti *et al.* [23]. Preparation of crude cell extracts and purification of TopI was prepared as previously described [24]. The protein concentrations were estimated from Coomassie blue-stained SDS-polyacrylamide gels by comparison to serial dilutions of BSA. Western blotting to test the expression level of TopI in cell extracts was performed essentially as described by Hede *et al.* [25].

### 2.3. Gel-Based Analysis of the RT-hTopI Sensor

The TopI-biosensor consists of two oligonucleotides (purchased from DNA-technology, Denmark), the L strand: 5′-AGA AAA ATT TTT ACA GGC CTA GC-C6amine and the Cl strand: 5′-GCT AGG CCT GTA AAA ATT T**T**T CTA AGT CTT TTA GAT CAT CGT TAT TCG ATG ATC TAA AAG ACT **TA**G A-BHQ1 where the bold underlined T was labeled with a 6-carboxyfluorescein (6-FAM), the bold underlined TA indicates the cleavage site, and the Black Hole Quencher 1 (BHQ1) was attached through a phosphothioate bond. Hybridization of the two oligonucleotides formed the active sensor.

**Figure 1.**
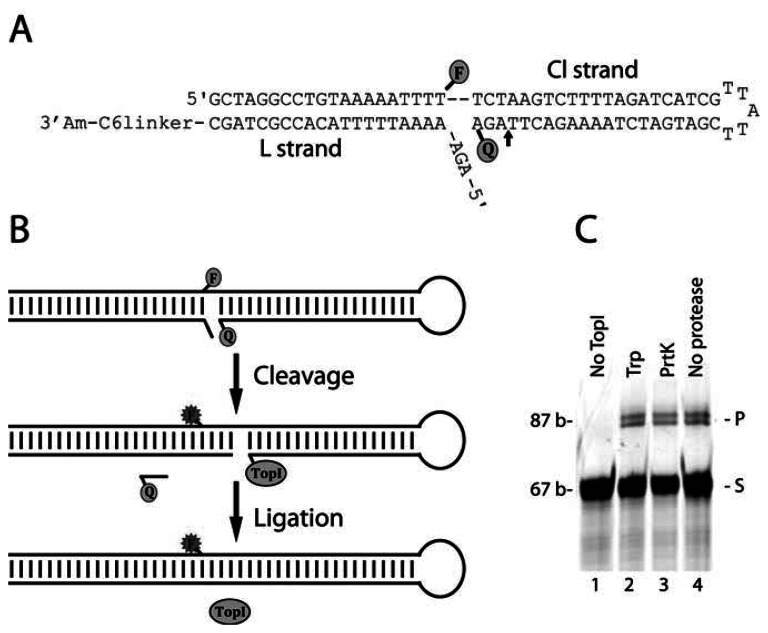
(**A**) Schematic illustration of the DNA sensor. The sensor is composed of a Cl strand that folds into a hairpin structure and contains an internal 6-FAM “F” and a 3′-BHQ1 “Q” moiety. The Cl is hybridized to a L strand with a 5′-OH end. The cleavage site is indicated by an arrow. (**B**) Flow chart of the anticipated reaction of hTopI with the DNA sensor. Cleavage results in release of the BHQ1 shifting the sensor to the ON state. Subsequently, ligation of the L strand releases hTopI and leaves the enzyme ready for another round of catalysis. The position of the fluorephore-quencher pair resulted in a quenching efficiency of approximately 80% (data not shown). (**C**) Gel picture showing the products resulting from incubating the DNA sensor with hTopI followed by trypsin- (lane 2) or proteinase K digestion (lane 3) or no protease treatment (lane 4). Lane 1 shows the gel-electrophoretic mobility of the unreacted sensor. The lengths (in bases) of the substrate and the upper product are indicted to the left of the gel-picture.

The sensor was prepared by incubating 50 pmol L strand and 25 pmol Cl strand in 1× TopI-buffer (10 mM Tris-HCl pH 7.5, 5 mM CaCl_2_, 5 mM MgCl_2_ and 0.1 mM DTT) for 5 min at 75 °C and cooled to room temperature. The reason for adding a surplus of L strand was to ensure that the more expensive fluorophore-quencher coupled Cl strands was hybridized with an L strand thus forming the sensor. Increasing the relative concentration of the L strand did not significantly affect the reactively of the sensor while decreasing the relative concentration of the L strand reduced the reactivity of the sensor as expected, since the effective concentration of fully annealed and active sensor decreased (data not shown). Subsequently, 600 fmol of purified hTopI enzyme was incubated with the sensor in a total volume of 20 μL containing 1× TopI buffer for 30 min at 37 °C. All reactions were terminated by the addition of SDS to a final concentration of 0.1% (w/v) and the samples precipitated by the addition of 300 mM NaCl and 3 volumes of 96% EtOH. Following precipitation the samples were redissolved in 1× TE-buffer and either left untreated, or digested with 1 mg/mL proteinase K or 0.1 mg/mL trypsin as indicated in Figure 1(C) following standard protocols. The samples were analyzed in a 12% denaturing polyacrylamide gel essentially as described by Christiansen *et al.* [26].

### 2.4. Real-Time Detection of TopI Activity

Activity measurements were carried out using a final concentration of 1 μM of DNA sensor in a volume of 50 μL containing 1× TopI buffer (10 mM Tris-HCl pH 7.5, 5 mM CaCl_2_, 5 mM MgCl_2_ and 0.1 mM DTT) and different concentrations of purified hTopI or CPT as stated in the individual figures. The components of the reactions were mixed at 4 °C before the reaction tubes were moved to a Bio-Rad iCycler IQ real-time PCR machine in which they were incubated at a constant temperature of 37 °C and data collected every 30 seconds for 5 h. The data was imported into excel and the initial reaction velocity extracted from the slope of the linear phase after the burst phase for each concentration of hTopI.

## 3. Results and Discussion

### 3.1. A Hairpin Shaped DNA Sensor Containing a Fluorophore-Quencher Supports Cleavage-Ligation Mediated by hTopI

The DNA sensor for measuring hTopI activity was composed of two DNA strands that formed a double-stranded DNA hairpin structure as outlined in Figure 1A. Readout was based on separation of a fluorophore (6-FAM)-quencher (BHQ1) pair placed on one DNA strand (named Cl in Figure 1(A)) of the sensor upon reaction with hTopI. In the OFF state of the sensor, 6-FAM and BHQ1 are brought into close proximity (facilitating approximately 80% quenching of 6-FAM (data not shown)) by the folding of the Cl strand into a hairpin having a protruding 5′-end (see Figure 1(A)). This results in BHQ1 and 6-FAM being positioned on each their side of the double helical stem of the hairpin and very close to the anticipated site of hTopI cleavage (indicated by an arrow in Figure 1(A)). Note that the design places both 6-FAM and BHQ1 in a region of the DNA sensor to which direct protein interaction is not a prerequisite for cleavage [26,27]. DNA binding and subsequent cleavage by hTopI upstream to the quencher moiety, leading to release of the quencher and emission of fluorescence light from 6-FAM, shifts the DNA sensor to the ON state (see flow chart in Figure 1(B)). To facilitate hTopI mediated cleavage and ligation a second DNA oligonucleotide was designed to hybridize to the protruding 5′-end of the DNA hairpin (the L strand shown in Figure 1(A)). This ensures the formation of double stranded DNA on both sites of the scissile phosphate (see Figure 1(A)), which has previously been demonstrated to be a prerequisite for hTopI-mediated cleavage [26]. Moreover, the 5′-OH end of the L strand presents a substrate for hTopI-mediated ligation [28] facilitating release of hTopI from the sensor, thus making another round of catalysis possible (see flow chart illustration, Figure 1(B)). The L strand contained a 3′-amine group enabling the potential attachment to a surface, although the current study has been demonstrated in a solution format.

First the interaction of hTopI with the sensor was investigated in a standard cleavage/ligation assay followed by denaturing gel-electrophoretic analysis of the products. For this purpose 600 fmol of hTopI was incubated with 25 pmol of sensor substrate for 30 min before the reactions were terminated by the addition of SDS and the reaction products either left untreated or treated with different proteases. By this, it is possible to determine whether potential products were covalently attached to a protein moiety *i.e*., hTopI, which would be the case for cleavage intermediates but not for products of ligation (see also Figure 1(B)). As evident from Figure 1(C) incubation of the sensor with hTopI resulted in two products, which were retarded in the gel relative to the sensor. These products were unaffected by treatment with different proteases or even omission of protease treatment prior to gel-electrophoresis, clearly demonstrating that they were unbound by protein and most likely result from ligation of the L strand of the sensor as outlined in Figure 1(B). The mobility of the largest of these products corresponded to 87 bases (see Figure 1(C)). This product size equals the expected product size resulting from cleavage three bases upstream of the quencher moiety of the Cl strand (which according to base sequence represents the preferred site of cleavage for hTopI) followed by ligation to the L strand. The slightly faster moving product most likely corresponds to cleavage one or two based upstream to the preferred site of cleavage followed by ligation. Such cleavage pattern has been reported previously for hTopI on different synthetic DNA substrates [26,28,29]. The cleavage products could not be observed directly, not even after inhibition of ligation by the addition of high concentrations of CPT or by 5′-phosphorylation of the L strand (data not shown), and are most probably scattered by the substrate band. Note, even after protease digestion the covalent attachment of hTopI to DNA results in gel-electrophoretic retardation of cleavage products due to the presence of a protease resistant peptide covalently attached to the DNA [26,28]. This may result in a mobility of the protease digested cleavage products close to the mobility of the quencher bound sensor.

### 3.2. Quantitative Measurement of hTopI Activity in Real-Time

**Figure 2.**
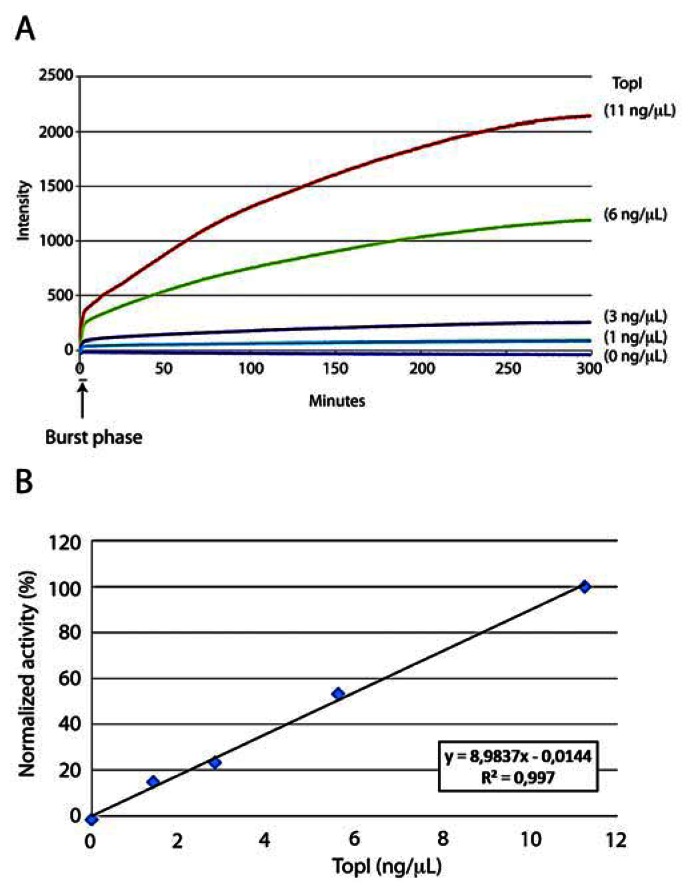
(**A**) A graphic depiction of the fluorescence emission measured as a function of time upon incubation of the DNA sensor with different concentrations of purified hTopI (indicated to the right of the figure) (**B**) Graphical representation of the results depicting the mean initial velocity of three individual reactions similar to the example shown in (A) plotted as a function of enzyme concentration. Data were normalized against the initial velocity of the highest enzyme concentration (corresponding to 100%) obtained in each repetition.

To investigate whether the DNA sensor could be used to detect hTopI activity quantitatively and in real-time, the sensor was incubated with serial dilutions of recombinant hTopI expressed in and purified to homogeneity from the yeast *S. cerevisiae* strain RS190. This strain lacks the endogenous TOP1 gene and has previously been used as a negative control for detection of hTopI with a DNA sensor [30]. The reactions were performed at physiological pH (pH 7.5) and in a standard hTopI buffer (see experimental section) with a final NaCl concentration in the reaction of 25 mM due to the presence of NaCl in the storage buffer of the purified hTopI. However, the sensor was tested to measure hTopI activity within the range 25–175 mM NaCl, which corresponds to the range of NaCl allowing hTopI mediated cleavage and within the pH range where hTopI is active (pH 6.5 to 8.5) (data not shown) [29]. The reactions were measured in real-time in terms of fluorescence emission in a qPCR machine. Note however, that since the reaction is isothermal any instrument capable of measuring fluorescence over time could be used. As evident from the graph shown in Figure 2(A), where fluorescence emission obtained in a representative experiment was plotted as a function of time, the sensor readily allowed the real-time measurement of hTopI activity in an enzyme concentration dependent manner, whereas incubation of the sensor with the catalytic inactive active site mutant, hTopI(Y723F), resulted in no increase in fluorescence (Supplementary Figure 1). The detection limit was approximately 0.04 ng/μL hTopI (Supplementary Figure 2(A)) and although this is less sensitive than a standard relaxation assay (Supplementary Figure 2(B)) or a rolling circle based detection [30] the presented assay is very different from the former mentioned assays since it allows real-time measurement and requires no additional amplification, staining, or gel-running. Thus, the data recorded is a direct real-time measurement of hTopI activity.

In order to ensure precise enzyme activity measurement (unaffected by substrate depletion and/or time-dependent bleaching of the 6-FAM fluorophore) the initial velocity calculated from the slope of the reaction curve in the initial linear phase observed immediately after a burst phase (indicated on the figure) was used as a measurement for enzyme activity (Figure 2(B)). The burst phase that was observed in this experiment indicates a potential rate limiting step after the first cycle of quencher release (resulting from the first cleavage reaction) and before the enzymes release the quencher in a second enzymatic cycle [31,32]. The molecular background for this behavior is yet unknown and requires further investigation. However, non-covalent DNA binding, which is a prerequisite for cleavage, is considered the rate-limiting step of TopI catalysis. Taking the rather bulky modifications of the presented DNA sensor relative to normal unmodified DNA into account we find it likely that non-covalent DNA binding after the initial round of reaction may cause a burst phase as the one observed. Further supporting this theory is the fact that the time lapse between mixing the components at 4 °C and initiating the measurements in the qPCR machine at 37 °C may leave time for the first round of non-covalent binding while the low temperature slows down cleavage that may then be initiated immediately after shifting the temperature to 37 °C. The next round of binding on the other hand may take time and slow down the reaction velocity which in turn may explain the occurrence of a burst phase.

As evident from Figure 2(B) depicting the mean of initial velocities of the reactions calculated from three individual experiments the sensor measures hTopI activity in a strictly concentration dependent manner with a direct and linear relationship between initial velocity and enzyme concentration. This is in agreement with the reactions following standard Michaelis-Menten kinetics [33].

### 3.3. The Hairpin-Shaped Sensor is Specific for TopI in Crude Biological Samples and Allows Measurement of hTopI Inhibition by CPT

As mentioned in the introduction hTopI is the cellular target for anti-cancer drugs of the CPT family, which convert the enzyme to a cell poison by selectively inhibiting the religation step of hTopI catalysis. Consequently, the cytotoxic effect of CPT correlates directly with the activity level and the CPT susceptibility of the cellular TopI. For the purpose of predicting chemotherapeutic response of individual patients easy means of measuring hTopI activity and drug susceptibility in clinical samples such as crude extracts from biopsies is therefore highly relevant.

**Figure 3.**
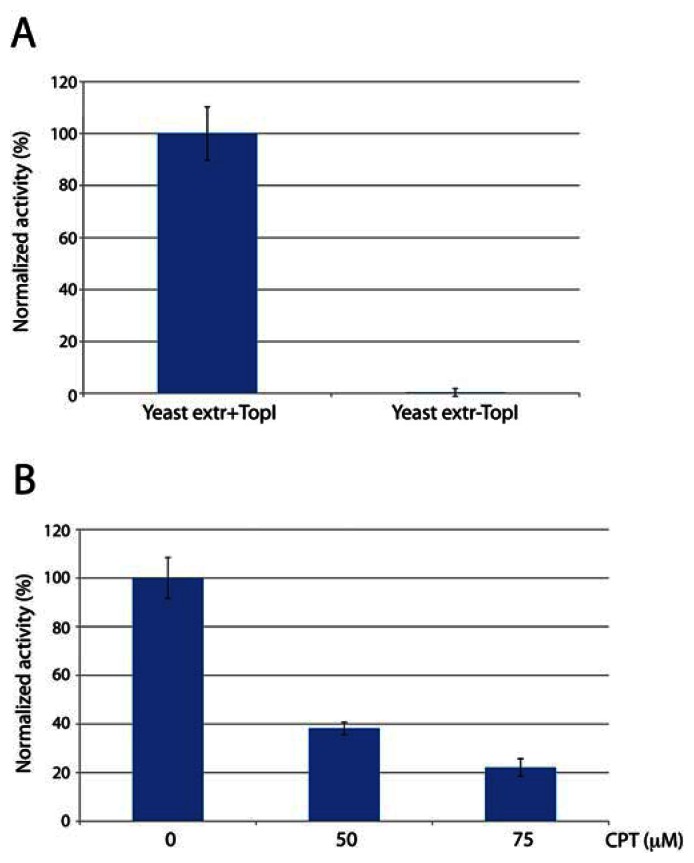
(**A**) Shows a bar chart depicting the initial velocity calculated from of three individual experiments where the DNA sensor were incubated with crude cell extracts from yeast *S. cerevisiae* expressing (Yeast extr+TopI) or not expressing (Yeast extr–TopI) hTopI. (**B**) Bar chart depicting the initial velocity of reactions performed in the absence or presence of 50 or 75 μM CPT. In both parts of the figure, data were normalized against the maximum initial velocity (corresponding to 100%) obtained in each repetition

In order to address the potential use of the DNA sensor for such purposes we investigated the specificity of the sensor towards hTopI in crude biological samples. As a model system we used whole cell extracts from the yeast strain RS190 (lacking the endogenous *TOP1* gene) with or without an episomal plasmid that supports the expression of recombinant hTopI. Following preparation of raw cell extracts, with or without hTopI activity, the extracts were incubated with the sensor and the reaction measured in terms of fluorescence emission in a qPCR machine. The initial velocity of the reaction observed in the two cell extracts were calculated as described for Figure 2 and the mean of the results obtained from three individual experiments depicted in Figure 3(A). The complete lack of activity observed in extract without hTopI strongly supports the specificity of the DNA sensor towards hTopI activity in raw cell extracts under the utilized reaction conditions.

In order to investigate the ability of the sensor to monitor inhibition of hTopI by anticancer drugs such as CPTs a fixed concentration (6 ng/μL) of hTopI was incubated with the DNA sensor in the presence of 0 to 75 μM of CPT and the activity measured in terms of fluorescence emission over time. The initial velocity of the reactions was calculated as described under Figure 2 and the mean of the results obtained from three individual experiments depicted as a bar chart in Figure 3(B). Consistent with previous reports the addition of increasing concentrations of CPT resulted in a reduced hTopI activity suggesting that the presented sensor may present an easy way of monitoring the drug response of intracellular hTopI extracted from various patient samples. Moreover, based on the ease by which activity measurement and drug response of hTopI can be performed using the DNA sensor, it may find use for future high-throughput drug screening programs aiming for identifying new hTopI targeting anticancer drugs.

## 4. Conclusions/Outlook

During recent years increasing numbers and designs of optical sensors for measuring different enzyme activities have been presented with the purpose of monitoring disease development and/or facilitate future individualized treatment protocols. Some of these are based on quantum dots or other artificial nano-particles, which present advantages such as enhanced photostability, easy multiplexed detection, improved sensitivity and temporal resolution [34,35]. However, the unique attributes of nanoparticle-based assays have in some cases been observed to affect the kinetics of enzymatic reactions making them less suitable for certain biological/clinical measurements in their current setups [36,37]. Hence, for biological relevant studies the combination of substrates designed to resemble the natural ones with small organic fluorophores/quenchers that minimize interference with the natural reactions may still be the best way to ensure precise measurements of both enzyme activities and potential drug interactions.

In the current study we present a quencher/fluorophore coupled DNA sensor allowing real-time and quantitative measurement of hTopI activity based on the release of a BHQ1 allowing fluorescence emission from the internally positioned 6-FAM of the sensor. The sensor is based on two oligonucleotides, which together form a double-stranded stretch of DNA, containing a preferred recognition sequence for hTopI. The quencher and fluorophore moieties are positioned to affect the hTopI activity as little as possible while still allowing the enzymatic transition of the sensor from the OFF to the ON stage. In line with this, the results of the activity measurements using the sensor were consistent with previously reported activity measurements of hTopI using more natural substrates and with the predictions of Michaelis-Menten kinetics. Furthermore the presented DNA sensor was specific towards hTopI even when assayed in crude cell extracts and allowed monitoring of the inhibition of hTopI activity by the anti-cancer drug CPT, which slows down the religation-step of hTopI catalysis by interacting specifically with the hTopI-DNA cleavage intermediate [13–15,].

hTopI is the sole cellular target of anti-cancer drugs of the CPT family that are currently used in the systemic treatment of colon-, ovarian- and small-cell lung cancers. However, it is far from all patients with these types of cancers that benefit from treatment with CPTs. The cytotoxic effects of CPTs have been demonstrated in several studies [10,16,17] to correlate directly with the intracellular activity level and CPT susceptibility of hTopI. We believe that the presented DNA sensor may provide an easy and fast way to monitor hTopI activity and CPT susceptibility in clinical relevant patient samples. Correlating the results of such studies with the patient response to CPT may pave the road for a highly improved and individualized treatment of cancer patients.

## Supplementary Material
