# Supplementary material for: DNA-Based Sensor for Real-Time Measurement of the Enzymatic Activity of Human Topoisomerase I
Source: Sensors (Basel). 2013 Mar 25;13(4):4017–28. doi: 10.3390/s130404017 (PMC3673067; doi:10.3390/s130404017)
Supplement: Supplementary file 1 [file sensors-13-04017-s001.docx]

**OPEN ACCESS**

sensors

**ISSN 1424-8220**

www.mdpi.com/journal/sensors

*Supplementary* *Information*

**DNA-Based Sensor for Real-Time Measurement of the Enzymatic Activity of Human Topoisomerase I. *Sensors* 2013, *13*, page range**

Lærke Bay Marcussen ^1,2,#^, Morten Leth Jepsen ^1,3,#^, Emil Laust Kristoffersen ^1,3,#^,
Oskar Franch ^1,3^, Joanna Proszek ^2^, Yi-Ping Ho ^3^, Magnus Stougaard ^2,3,^* and
Birgitta Ruth Knudsen ^1,3,^*

^1^ Department of Molecular Biology and Genetics, Aarhus University, Aarhus C 8000, Denmark;
E-Mails: laerke_bay@hotmail.com (L.B.M.); mortenlethjepsen@gmail.com (M.L.J.); emillk@mb.au.dk (E.L.K.); oskar.franch@post.au.dk (O.F.)

^2^ Department of Pathology, Aarhus University Hospital, Aarhus C 8000, Denmark;
E-Mail: joanna.proszek@gmail.com

^3^ Interdisciplinary Nanoscience Center (iNANO), Aarhus University, Aarhus C 8000, Denmark;
E-Mail: megan.ypho@inano.au.dk

**^#^** These authors contributed equally to this work.

***** Authors to whom correspondence should be addressed; E-Mails: magnstou@rm.dk (M.S.); brk@mb.au.dk (B.R.K.).

Contents

**Figure S1.** Shows a bar chart depicting the initial velocity calculated from three individual experiments where the DNA sensor was incubated with either no hTopI or 2.5 ng/μL purified hTopI (Y327F) lacking the active site tyrosine or 2.5 ng/μLwild type hTopI. The initial velocity of the wild type enzyme was normalized to 100%.

**
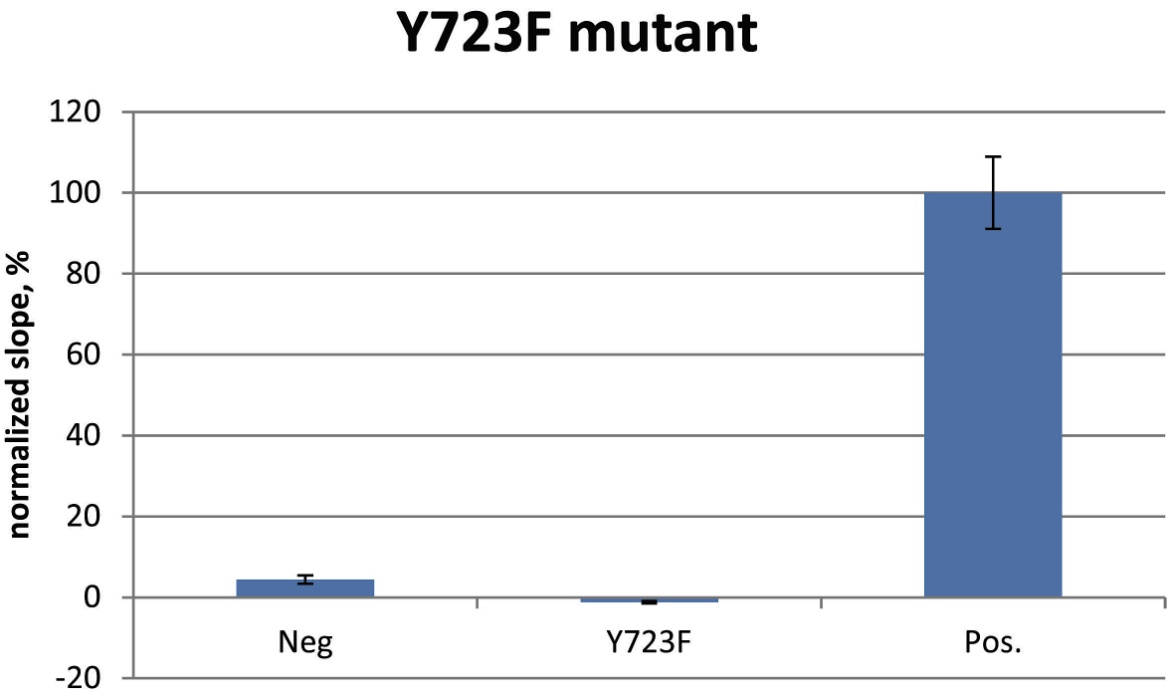
**

**Figure S2.** (**A**) Shows a bar chart depicting the initial velocity calculated from three individual experiments where the DNA sensor was incubated with decreasing concentrations of hTopI as indicated in the figure. (**B**) Shows the result of a relaxation experiment where 200 ng of supercoiled pUC19 was incubated with 2.500, 1.250, 0.625, 0.208, 0.042, 0.021, 0.008, or 0.004 ng/μL (lanes 2–9) of hTopI for 30 minutes at 37 °C in a 1× TopI buffer containing 150 mM NaCl, which is optimal for hTopI mediated relaxation. Lane 1 is a negative control with supercoiled plasmid incubated without hTopI. The lower band represents supercoiled plasmid. The upper band represents fully relaxed plasmid and the intermediate bands represent partly relaxed plasmid.

**
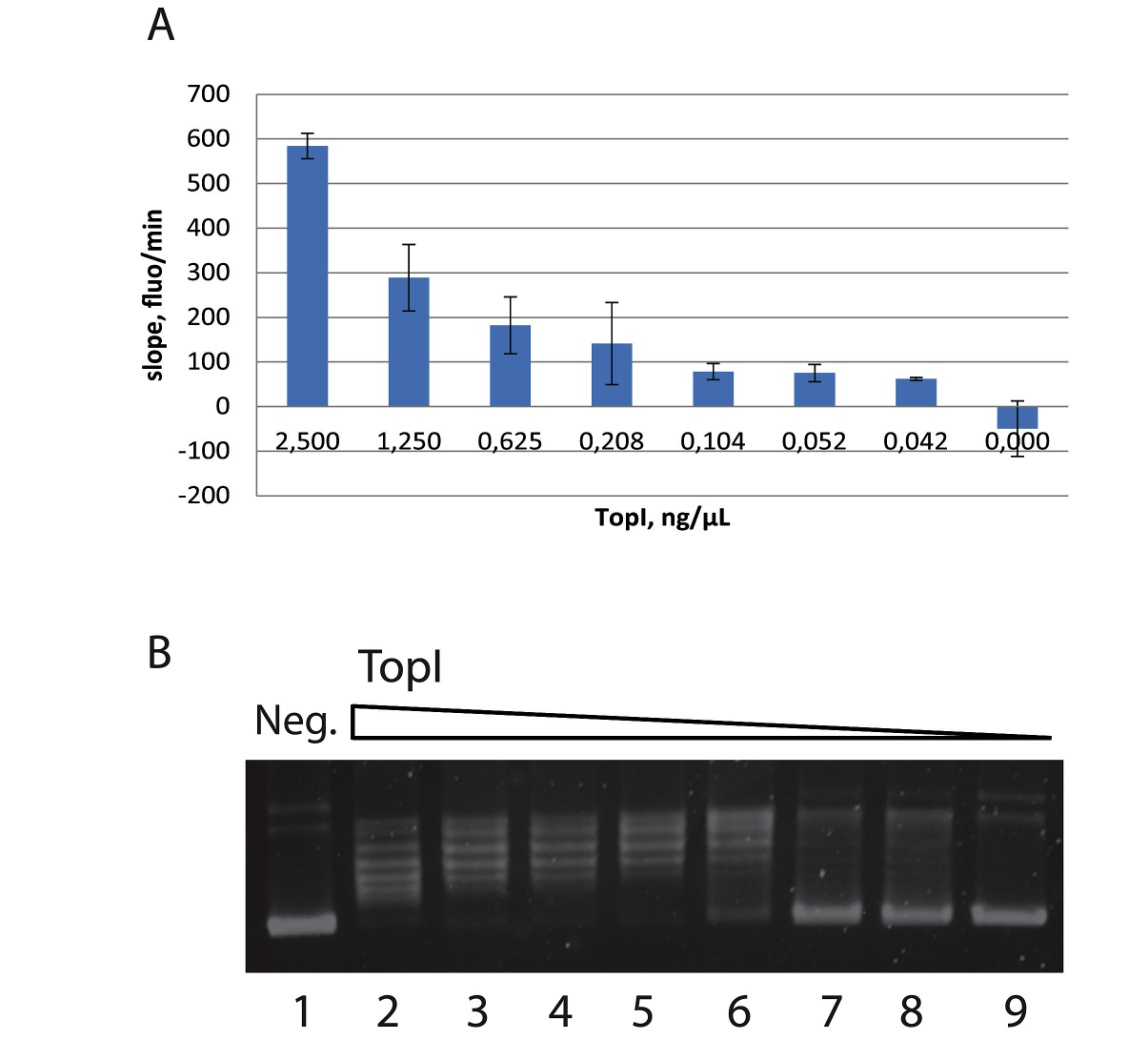
**

© 2013 by the authors; licensee MDPI, Basel, Switzerland. This article is an open access article distributed under the terms and conditions of the Creative Commons Attribution license (http://creativecommons.org/licenses/by/3.0/).
